# A study of Docetaxel-induced effects in MCF-7 cells by means of Raman microspectroscopy

**DOI:** 10.1007/s00216-012-5887-9

**Published:** 2012-03-08

**Authors:** Katharina Hartmann, Melanie Becker-Putsche, Thomas Bocklitz, Katharina Pachmann, Axel Niendorf, Petra Rösch, Jürgen Popp

**Affiliations:** 1 Institute of Physical Chemistry and Abbe Center of Photonics, University of Jena, Helmholtzweg 4, 07743 Jena, Germany; 2 Institute of Photonic Technology, Albert-Einstein-Straße 9, 07745 Jena, Germany; 3 Department of Hematology and Oncology, Clinic for Internal Medicine II, University Hospital Jena, 07740 Jena, Germany; 4 Pathologie Hamburg-West, Institut für diagnostische Histopathologie und Zytologie, Lornsenstraße 4, 22767 Hamburg, Germany

**Keywords:** Raman microspectroscopy, Docetaxel, Breast cancer, MCF-7

## Abstract

Chemotherapies feature a low success rate of about 25%, and therefore, the choice of the most effective cytostatic drug for the individual patient and monitoring the efficiency of an ongoing chemotherapy are important steps towards personalized therapy. Thereby, an objective method able to differentiate between treated and untreated cancer cells would be essential. In this study, we provide molecular insights into Docetaxel-induced effects in MCF-7 cells, as a model system for adenocarcinoma, by means of Raman microspectroscopy combined with powerful chemometric methods. The analysis of the Raman data is divided into two steps. In the first part, the morphology of cell organelles, e.g. the cell nucleus has been visualized by analysing the Raman spectra with *k*-means cluster analysis and artificial neural networks and compared to the histopathologic gold standard method hematoxylin and eosin staining. This comparison showed that Raman microscopy is capable of displaying the cell morphology; however, this is in contrast to hematoxylin and eosin staining label free and can therefore be applied potentially in vivo. Because Docetaxel is a drug acting within the cell nucleus, Raman spectra originating from the cell nucleus region were further investigated in a next step. Thereby we were able to differentiate treated from untreated MCF-7 cells and to quantify the cell–drug response by utilizing linear discriminant analysis models.

**Electronic supplementary material:**

The online version of this article (doi:10.1007/s00216-012-5887-9) contains supplementary material, which is available to authorized users.

**Figure:**
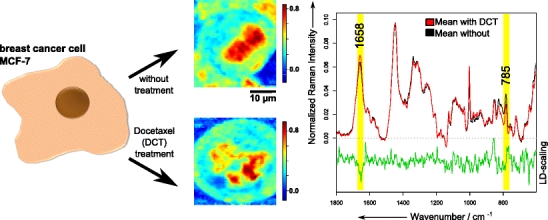
Raman microspectroscopy in combination with powerful chemometric methods (e.g. artificial neural networks) indicates morphological (nucleus fragmentation) and spectral changes in Docetaxel treated breast cancer cells (MCF-7) in comparison to untreated cell samples

## Introduction

Breast cancer is the most common cause of death of women worldwide [1]. Due to the low effectiveness of chemotherapeutic agents of about 25% as one possible origin of the high mortality rates [2], it is of utmost importance to achieve a comprehensive understanding about the therapeutic mechanism of cytostatics in order to improve chemotherapy. In addition, monitoring the efficiency of an ongoing chemotherapy and supporting the choice of the most effective cytostatic drug would be an important step towards personalized therapy.

In the current clinical practice, the choice of chemotherapeutic drugs for patients is based on clinical trials and observations made on the courses of a disease for other patients. This practice harbours the risk that the individuality of patients is disregarded. Also the selection of drug combinations is widely empirical and often done by trial and error. However, since the introduction of chemotherapeutic agents investigations deal with an optimization of the drug selection for the individual patient. Especially in vitro chemosensitivity tests to predict a chemotherapy response gain impact, e.g. 3-(4,5-dimethyl-thiazol-2-yl)-2,5-diphenyl-tetrazolium bromide (MTT), membrane and ATP assays and genomic analyses [3–5]. Nevertheless, some assays turned out to be insufficient of directing therapy [5] and the above mentioned techniques are not widely used in clinical practice.

During the last decades Docetaxel (DCT, Taxotere®) became a widely used chemotherapeutic agent [6] against prostate, lung, stomach and ovarian cancer. It is known as highly active anticancer drug and one of the most effective medication against breast carcinoma [7]. DCT is a semi-synthetic taxane, which is produced by the esterification of 10-deacetyl baccatin III, an extract from needles of the European yew (*Taxus baccata* L.) [8].

DCT has an enoumerous affinity to the β-tubulin of the microtubules of the cytoskeleton. Commonly there is an equilibrium between polymerisation and depolymerisation of the microtubles. This dynamic reorganisation is import for several cellular processes, e.g. the uptake, transport and secretion of vesicles, cell movements, formation of the cell shape and segregation of the chromosomes during mitosis [9]. A DCT treatment disturbs the depolymerisation of the microtubles causing a cell-cycle arrest [9–11]. Thereby, an ongoing controversy about the exact mode of action and the death mechanism exists: Morse et al. determined the non-apoptotic mitotic catastrophe with its missegregation of the chromosomes during mitosis and resulting micronuclei as the primary death mechanism of DCT treated human breast cancer cells, e.g. MCF-7 [9]. In contrast several other contributions report about apoptosis as the main mechanism of cell death after a DCT treatment induced mitotic catastrophe [12–15]. In this controversy, it should be also considered, that the response of antimicrotuble drugs might cause a dose- and cell line-specific mixtures between apoptotic and mitotic cell death, depending on the genetic background [9, 10, 16].

Raman spectroscopy in combination with optical microscopy has established itself because of its non-invasive character, high molecular specificity, minimal sample preparation and high spatial resolution in the micrometer range to an extremely powerful analytical method. Thereby Raman microscopy has been recognized within the last years to be a very capable method to analyse biological samples like, e.g. eukaryotes [17–21] as well as prokaryotes [22–28] and even viruses [29]. By doing so, it allows answering a broad range of biomedical questions like, e.g. early diagnosis of cancer [30–36]. Because of the subtle biological differences involved, this requires a multivariate treatment of Raman spectra in order to extract all necessary bio-chemical information [37].

In comparison to Fourier transform infrared spectroscopy, which is also capable to acquire structural information of cell organelles [38], Raman microspectroscopy provides the unique capability to observe biochemical information from living cells [39–41]. Thereby, the non-disturbing low Raman-scattering cross section of water is of crucial importance. It allows the investigation of living cells in buffer solution or in their culture media.

Furthermore, several publications deal with the effects of chemotherapeutic agents on cells: In 1982, Manfait et al. already investigated the interaction of the anthracycline Adriamycin with human DNA via Raman and resonance Raman spectroscopy and found that the chromophores of Adriamycin intercalate into the GC sequences of the DNA and that hydrogen bonds are formed [42]. In recent years, many experiments cover the biochemical and spectral changes in Raman spectra of cells undergoing apoptosis due to a treatment with chemotherapeutic drugs. Especially the investigation of living cells gains impact [39–41]. Thereby, Raman spectra from the same living cells at different intervals were detected. Zoladek et al. even presented an experimental setup, which allows the maintenance of the cells under sterile physiological conditions during Raman measurements [40]. Currently, the optimization of the experimental design is of interest. Laser tweezers were applied to both optical trapping of single suspensions cells and simultaneous Raman spectroscopy [43] as well as single-cell patterned microarrays [39]. In addition, modern techniques like resonance Raman and surface enhanced resonance Raman scattering [44] and surface enhanced Raman scattering (SERS) [45] were utilized to monitor the effects introduced by chemotherapeutic agents on cancer cells. Hence, SERS has shown its potential as a simple and sensitive drug screening tool potentially even in vivo.

The aim of the current study is to investigate the interaction between DCT and the cultivated human breast adenocarcinoma cell line MCF-7 by Raman microspectroscopy in combination with special statistical/chemometrical methods. The resulting Raman images will be validated by the histopathological gold standard hematoxylin and eosin staining. Furthermore the additionally information in the Raman scans should be used for determining the function of DCT on MCF-7 cells in a quantitative and objective manner. The successful realization of these goals would be a significant step towards a personalized medicine applying Raman microspectroscopy as control tool.

## Materials and methods

### Cell line selection and cell cultivation

The breast cancer cell line MCF-7 (Michigan Cancer Foundation-7), established by Soule et al. in 1973, contains isolated epithelial cells from a pleural effusion from a 69 years old caucasian female patient with metastatic mammary carcinoma [46]. Cells were cultivated in cell culture flasks with Roswell Park Memorial Institute 1640 medium supplemented with 10% fetal bovine serum and 1% penicillin/streptomycin. Cultures were maintained at 37 °C in a humified atmosphere of 5% CO_2_. For the experiments, cells were harvested by trypsinization, centrifugation and finally re-cultured on fused silica slides (Frank Optic Products GmbH, Germany) in petri dishes with the same cultivation conditions for further 24 h.

### Cell treatment with DCT

DCT powder was resolved in ethanol and diluted by the growth media. Thereby, the used DCT solution contains an ethanol content of only 0.1% to prevent chemical alterations in MCF-7 cells.

MCF-7 cells were exposed to the growth medium supplemented with various DCT concentrations. Table 1 gives an overview of the applied concentrations and exposure times. In the first test series, slides with grown cells were treated with 10 and 100 nM DCT concentration for 24 h (Batch 1) and 48 h (Batch 2), respectively. The cells of Batch 3 were incubated for 24 h with DCT. Subsequently the DCT containing medium was replaced with a normal medium (without DCT). A cell cultivation step of further 24 h followed. The second test series (Batch 4) contained MCF-7 cells treated for 48 h with DCT concentrations of 2.5, 5 and 7.5 nmol/l. Cultures of untreated cells were used as references. Each test series (batch) was prepared twice. For Raman data acquisition, the cells were air dried and stored at room temperature. For each batch, 2 to 6 cells were measured, while approximately 20,000 Raman spectra were recorded.

**Table 1:** Overview of applied DCT treatments

Batch	Slide number	Exposure time (h)	DCT concentration (nmol/l)
First test series			
1	T01	24	0
	T02		10
	T03		100
2	T04	48	0
	T05		10
	T06		100
3	T07	24 + 24	0
	T08		10
	T09		100
Second test series			
4	T10	48	0
	T11		2.5
	T11		5
	T11		7.5
	T12		10
	T13		100

### Hematoxylin and eosin staining

Hematoxylin and eosin (H&E) staining was used as common histopathological analysis for comparison with the Raman data. H&E staining allows the assignment of various structures in tissues and single cells [47]. Hematoxylin stains all basophilic components blue, especially the nucleus containing DNA and RNA, and the rough endoplasmatic reticulum with an accumulation of ribosomes as a result of a coordination bond between aluminum and phosphor atoms of the DNA and RNA [48]. Eosin stains the cytoplasm, connective tissue and collagen fibers (eosinophilic substances) red due to ionic bonds between the anionic dye and cationic plasma proteins. The samples were stained according to a well established standard protocol [49].

### Raman microspectroscopy

Raman measurements were performed with the confocal Raman microscope [50] CRM 300 (WITec, Germany) equipped with a 50×/NA 0.95 objective (Zeiss, Germany). As excitation light, the 785 nm output of a diode laser was used with a power of 50 mW at the sample. For each spectrum, an acquisition time of 10 s was applied. The back scattered light was spectrally dispersed with a monochromator of 300 nm focus length equipped with a 600 lines/mm grating and detected by an EM-CCD camera with pixels operated at −75 °C. The cell Raman maps were recorded with a step size of 0.5 μm.

### Chemometric evaluation

The large amount of Raman data was evaluated applying chemometric methods. The calculations were performed on a commercially available PC system (Intel(R) Core(TM) 2Duo CPU, E67502.66 GHz, 1.97 GB RAM). The computations were done in R [51], a statistical language. All Raman spectra were preprocessed as described in a previous publication [32], while here only a brief overview is given.

First a wavenumber calibration employing the commonly used wavenumber standard 4-acetamidophenol was performed. For the chemometric analysis, the Raman spectra were truncated to the wavenumber region between 600 and 1,800 cm^−1^ (part of the fingerprint region). Afterwards, a background subtraction was performed applying a 5th order polynomial [52] to correct for the fluorescence background followed by a vector normalization. Prior to the analysis, a dimension reduction to 50 PCA scores was carried out to reduce the dimension of the data matrix without losing too much information. The chemometric analysis includes *k*-means cluster analysis [53], artificial neural networks (ANN) [32] and linear discriminant analysis (LDA) models [54]. While the *k*-means cluster analysis and the ANN were used for visualization, the LDA model was applied as a classification technique. The LDA was cross-validated by a leave-one-out-cross-validation (LOO-CV) [55].

## Results and discussion

### Visualization of the cell morphology—qualitative analysis

DCT induces nucleus fragmentation due to apoptosis or mitotic catastrophe at the beginning of the effect chain [9–11, 16]. For that reason, the first aim was the visualization of the morphology of MCF-7 cells. This will be an important step for further analyses, e.g. for the realization of a quantitative analysis presented in the “Determining the cell–drug response—quantitative analysis” section. In order to visualize the cell morphology, we applied an ANN to analyse the Raman *xy*-scans of whole single cells. The ANN analysis yields the DNA/RNA distribution for each measured Raman spectrum allowing the creation of a Raman image displaying the DNA/RNA content of the whole cell. These DNA/RNA Raman maps can be validated by comparing them with the histopathologic gold standard H&E staining. This validation of the applied ANN algorithm showed that DNA/RNA Raman maps display the position and shape of the cell nucleus.

**Fig. 1:**
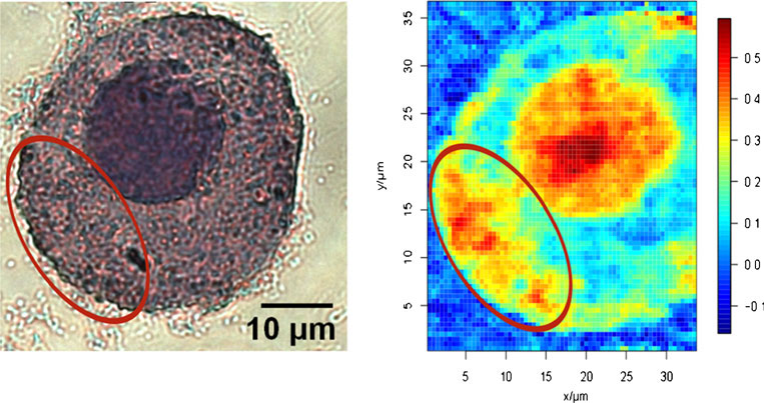
Comparison of a MCF-7 cell stained with H&E (*left*) and the chemical map of the DNA/RNA distribution based on Raman spectroscopic data (*right*). The *circle* marks a structure in the cell with further DNA or RNA content outside the nucleus

Figure 1 compares the DNA/RNA distribution generated by an ANN with a subsequently stained H&E image of the same non DCT treated cell. In the Raman image, high DNA/RNA concentrations are displayed in red, while low concentrations appear blue. It can be clearly seen that the position, shape and size of the nucleus are comparable in both images. Furthermore, the lower left region of the cell (see encircled region in Fig. 1) shows one further structure both visible in the H&E as well as in the image generated by an ANN. The origin of this cannot be definitely clarified, but one possibility could be the presence of extra nuclear RNA, e.g. rough endoplasmatic reticulum or messenger, transfer or ribosomal RNA. This assumption was confirmed by Figure S1 in the Electronic Supplementary Material displaying Raman spectra from the nucleus and cytoplasm. The Raman spectra of cytoplasm and the nucleus show a significant Raman band at 811 cm^−1^ assigned to the vibration of the sugar-phosphate backbone of the RNA [56]. Therefore, the presence of extra nuclear RNA is probable in the nucleus as well as in the cytoplasm. Overall, Raman microspectroscopy provides similar information as the histological gold standard H&E staining. The great advantage is that Raman microspectroscopy is label free and therefore potentially capable for in vivo applications in comparison with the H&E stain. This proof-of-principle is necessary for further experiments dealing with the effect of docetaxel on living cancer cells over a longer period of time. To investigate such dynamic cellular events, a measurement technique requiring a label is not suitable. Raman microspectroscopy is label free and non-invasive and thus, cellular functions remain untouched in living cells during the measurement. Overall, the used ANN algorithm is ideally suited to identify Raman nuclei spectra, which can be further analysed (see the “Determining the cell–drug response—quantitative analysis” section).

**Fig. 2:**
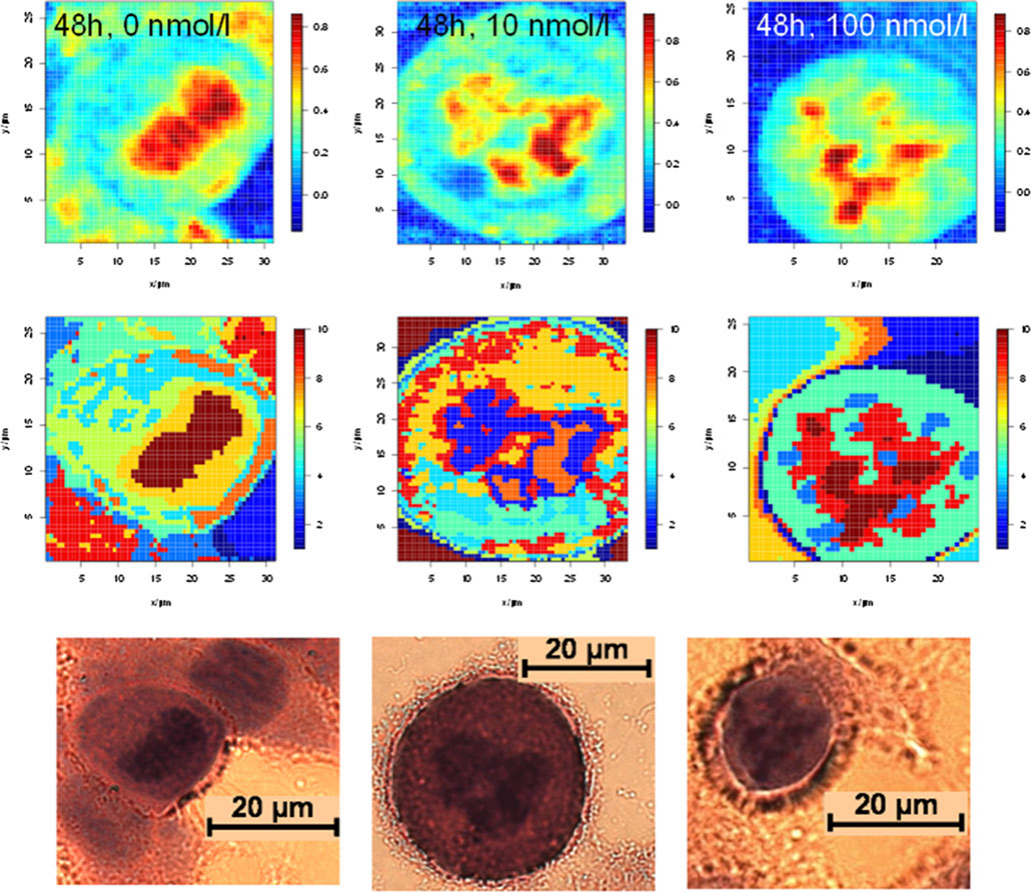
Raman images of whole single cells generated by ANN analysis showing the DNA/RNA distribution of untreated (0 nmol/l) and DCT treated (10 nmol/l, 48 h and 100 nmol/l, 48 h) MCF-7 cells. *Dark red regions* mark high DNA/RNA concentrations and thereby the nucleus position (*1st row*); results of the *k*-means cluster analysis (*2nd row*) and corresponding H&E images of the same cells (*3rd row*)

In the following the ANN Raman images will be analysed with respect to identify structural differences in treated and untreated MCF-7 cells. Figure 2 (first row) depicts the Raman images of the DNA/RNA content of MCF-7 cells treated with 0, 10 and 100 nmol/l DCT for 48 h. In addition, *k*-means cluster analyses were carried out to visualize cell compartments, mainly the nucleus region (Fig. 2, second row). Both chemometrical methods (ANN and *k*-means cluster analysis) generated the same result: untreated (0 nmol/l) MCF-7 cells show compact cell nuclei, while the cells treated with 10 nmol/l as well as with 100 nmol/l DCT for 48 h exhibit fragmented nuclei. These pronounced differences between treated and untreated cells were confirmed by subsequent H&E staining of the same samples (Fig. 2, third row).

Moreover, DCT induced effects on the cell morphology become visible in almost all measured MCF-7 cells. Some examples are shown in Figure S2 in the Electronic Supplementary Material. The Raman images illustrate once more an ANN calculation displaying the DNA/RNA content. These cells were treated with 100 nmol/l DCT for the three different exposure times of 24 h, 48 h and 24 + 24 h (see Table 1). It can be clearly seen that almost all MCF-7 Raman images are characterized by fragmented nuclei. An exception from this behavior can be found for cell 2C showing a homogeneous cell nucleus, which might be attributed to the fact that DCT is only effecting cells during mitosis. Since the cell cycle was not synchronized, it is possible that the cell 2C was not in a mitotic stage. Furthermore, the individual behavior of a cell should be also considered.

However, these results point out that Raman microspectroscopy combined with chemometrical analysis has a great potential to image chemical changes in cells. The used ANN algorithm is an optimal method to visualize the cell nuclei and thereby identify Raman nuclei spectra. The identification of nuclei spectra is an important step result for the subsequent quantitative analysis of the cell–drug response for DCT treated and untreated MCF-7 cells.

### Determining the cell–drug response—quantitative analysis

It is of utmost importance to improve and personalize chemotherapy. Thereby the appropriate choice of the most effective chemotherapeutic drug for the individual patient and the possibility of an online monitoring of the progress of the chemotherapy are two of the major challenges towards increasing the effectiveness of cytostatic drugs and towards a personalized treatment. Raman microspectroscopy in combination with chemometrical methods has the potential to significantly contribute to tackle these challenges. Raman images in contrast to H&E images are of multivariate nature that is they contain additional spectral information which can be used to differentiate between untreated and treated MCF-7 cells or to unravel the (spectral) cell–drug response.

**Fig. 3:**
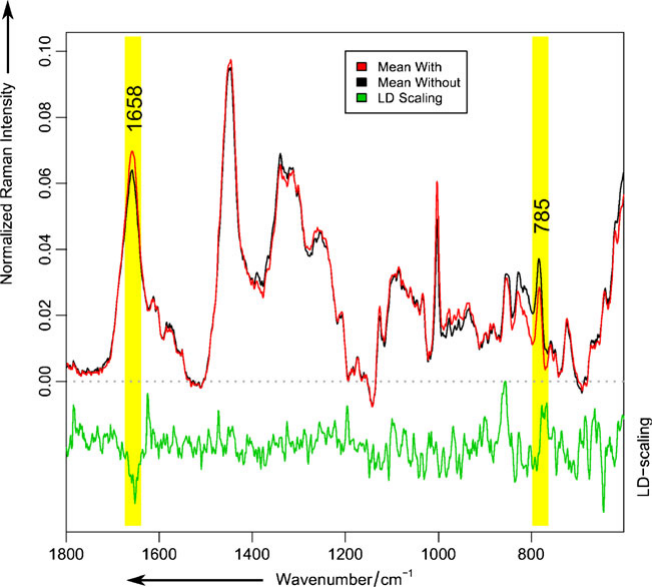
Raman mean spectra of DCT treated (*red spectrum*) and untreated (*black spectrum*) MCF-7 cells. Significant differences are visible, e.g. the DNA/RNA peak at 785 cm^−1^ and amide I peak at 1,658 cm^−1^. These regions are confirmed by the LD scaling vector (*green spectrum*)

In order to differentiate between untreated and DCT treated MCF-7 cells, a LDA was utilized to classify the nucleus Raman spectra of treated and untreated cells. The resulting classification model was evaluated by a LOO-CV and the confusion matrix is given in Table 2. The LDA model classified 10,176 out of 10,253 cell nucleus Raman spectra of treated cells and 8,294 out of 8,356 cell nucleus Raman spectra from untreated cells correctly, which corresponds to an accuracy of 99.2%. This accurcy rate together with the achieved sensitivity and specificity of also 99.2% shows that Raman microspectroscopy is perfectly suited to differentiate between treated and untreated MCF-7 cells. Figure 3 displays the mean Raman spectra of the two classes of the LDA model that is DCT treated (red) and untreated (black) MCF-7 cells together with the corresponding LD scaling vector. The LD scaling vector (green) indicates the spectral differences within the Raman spectra allowing for the clear separation between treated and untreated cells. A comparison between the mean Raman spectra of treated and untreated MCF-7 cells show that the intensity of the band at 785 cm^−1^ which can be assigned to a DNA/RNA vibration (OPO stretch DNA/RNA) [56, 57] is increased in the mean Raman spectrum of DCT treated cells as compared to untreated cells. The reason for this increasing 785 cm^−1^ band intensity could be a local DNA concentration enhancement due to the nucleus fragmentation and the corresponding increased DNA condensation. Furthermore the amide I vibration at 1,658 cm^−1^ [56, 57] is decreased in intensity in the mean Raman spectrum of treated MCF-7 cells as compared to the spectrum of untreated cells, which might be also a consequence of the nucleus fragmentation and the corresponding degradation of the protein structures in the nucleus.

**Table 2:** Confidence table of the LDA model for separation of control data vs. Raman data containing cell–drug interactions

Predicted labels	True labels	
	Raman spectra of treated cells	Raman spectra of untreated cells
Raman spectra of treated cells	10176	62
Raman spectra of untreated cells	77	8294

**Fig. 4:**
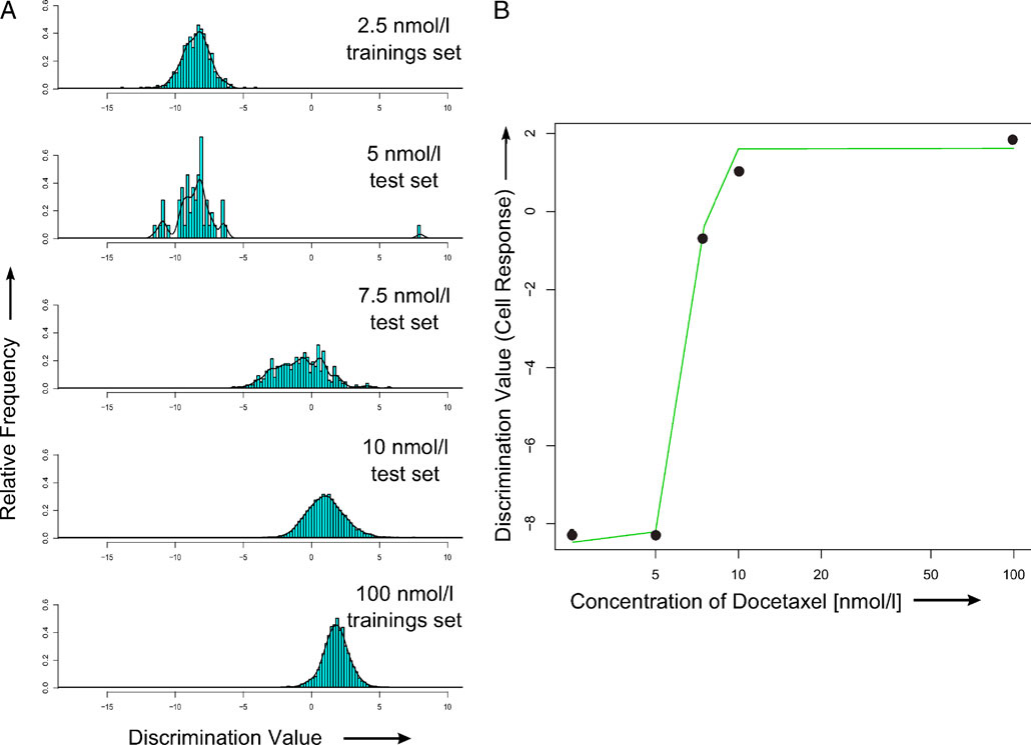
(**A**) LDA classification of Raman data of various DCT treatments. The histogram of the LD value (scalar product) of all five DCT concentrations is given. With the lowest and highest concentration, the LDA model was trained and all spectra were projected on the LDA model. (**B**) The dose–effect relationship, as a function of the logarithmically DCT concentration, is visualized. The LD-value can be interpreted as cell response for DCT treatment and shows a sigmoidal trend, which can be utilized for determining the spectral detection limit

In order to investigate DCT concentration dependent cell-response Raman spectra recorded for cells treated with 2.5, 5, 7.5, 10, and 100 nmol/l DCT for 48 h were analysed (see Table 1). It should be noted that only the Raman spectra originating from the cell nucleus were considered. A LDA model was used to calculate the spectral differences between the five different concentrations. The LDA model was trained with two training sets including Raman spectra of cells exposed to DCT concentrations of 2.5 and 100 nmol/l for 48 h to achieve the corresponding LD scaling vector. As test sets, Raman spectra of cells treated with 5, 7.5, and 10 nmol/l DCT were projected on this LD scaling vector. Figure 4A gives an overview of the drug concentration dependent class affinities. Here, the distribution of the scalar product of each Raman spectrum with the calculated LD vector is shown (LD value). Overall, the LDA analysis reveals that the highest cell response occurs after a treatment with DCT concentrations between 7.5 and 10 nmol/l showing that the lowest concentration that is detection limit where the influence of DCT on the MCF-7 Raman spectra is still visible lies at a DCT concentration of 7.5 nmol/l. In Fig. 4B, the LD value (e.g. the inner product of every spectrum with the LDA value) is plotted against the drug concentration on a logarithmic scale. This LD value can be interpreted as the cell response. The points in Fig. 4B result from the maxima of the histogram in Fig. 4A for every DCT concentration. The arrangement of the values suggests a sigmoidal trend, which confirms studies about the dose-effect relationship by Jodrell et al. [58] and Jakobsen et al. [59]. These findings indicate, that even low DCT concentrations of 7.5 nmol/l induce chemical alterations in MCF-7 cells.

## Conclusion

We investigated DCT induced effects on a breast cancer model system (MCF-7) by means of Raman microspectroscopy in combination with powerful chemometrical methods. DCT induces nucleus fragmentation at the beginning of the effect chain. Therefore, we visualized the morphology of cell organelles, e.g. the cell nucleus by applying *k*-means cluster analysis and an ANN. The ANN generated images are highly correlating with the DNA/RNA content. The in that way generated Raman images highlighted DCT induced morphological alterations in the nuclei structure, caused by the drug induced depolymerisation of microtubules during mitosis. These images based on Raman spectra were validated with the histopathologic gold standard method, the H&E staining. Overall, both Raman imaging analysing methods (*k*-means clustering and ANN) were capable of visualization the cell morphology.

By using a LDA with the preselected nuclei spectra, it was possible to differentiate between treated and untreated MCF-7 cells with an accuracy of 99.2%. Thereby, Raman mean spectra of DCT treated and untreated cells show significant differences at 785 cm^−1^, which can be assigned to a DNA/RNA vibration, and the amide I peak at 1,658 cm^−1^ due to the DCT induced fragmentation of the nuclei. DCT treatments with concentrations lower than 10 nmol/l (2.5, 5 and 7.5 nmol/l) were implemented additionally to achieve quantitative results about the efficiency of DCT by applying a LDA analysis. A drug concentration of 7.5 nM was indicated as the detection limit of monitoring spectral changes in Raman spectra of treated MCF-7 cells.

In the present contribution, it was shown that the analysis of cellular Raman spectra by means of modern chemometric approaches is capable of monitoring the impact of cytostatic agents. The effect of a drug on the cell morphology can be visualized and the spectral differences between treated and untreated cells can be calculated. With the help of the visualization and detection of the effect of chemotherapeutic drugs it could be possible to monitor the ongoing chemotherapy. This could be achieved by applying the presented methodology on real patient cell samples. In that way, the process and the benefit of an ongoing chemotherapy would be determined in an objective manner. Therefore an individualized medicine with Raman spectroscopy as control tool seems reasonable. The above presented results can be used in another way. If quantum mechanical calculations (based on DFT calculations or in the Hartree–Fock regime) [60, 61] of DCT, microtubules and the respective motor proteins are carried out, a deeper understanding of the work mechanism of DCT can be obtained. This workflow is outlined in [62, 63]. Nevertheless, the quantum mechanical calculations have to be optimized for the application to huge proteins and cell structures. This is an emerging field, which in combination with experimental data, like presented above, would enhance our understanding of the bio-chemical processes inside of cells and the their interaction with drugs.

## Electronic supplementary material

Below is the link to the electronic supplementary material.

ESM 1 (PDF 398 kb)
