# Supplementary material for: A study of Docetaxel-induced effects in MCF-7 cells by means of Raman microspectroscopy
Source: Anal Bioanal Chem. 2012 Mar 8;403(3):745–53. doi: 10.1007/s00216-012-5887-9 (PMC3336052; doi:10.1007/s00216-012-5887-9)
Supplement: Supplementary file 1 — (PDF 398 kb) [file 216_2012_5887_MOESM1_ESM.pdf]

Analytical and Bioanalytical Chemistry

Electronic Supplementary Material

A study of Docetaxel-induced effects in MCF-7 cells by means of Raman microspectroscopy

Katharina Hartmann, Melanie Becker-Putsche, Thomas Bocklitz, Katharina Pachmann, Axel Niendorf, Petra Rösch and Jürgen Popp

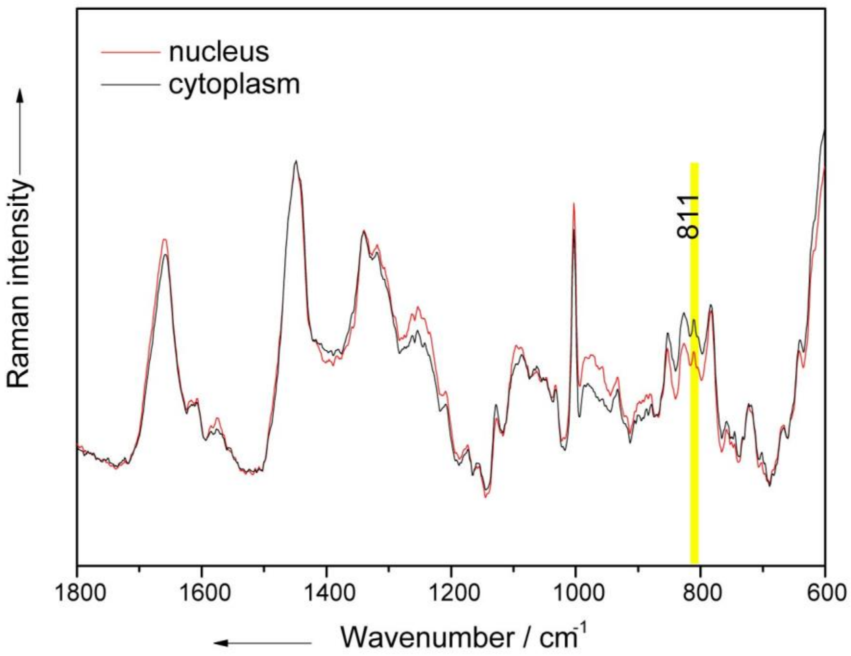

Figure S1: Raman spectra of cytoplasm (black) and nucleus (red). Significant Raman band at 811 cm^{-1} assigned to the vibration of the sugar-phosphate backbone of the RNA [56] is clearly visible in both spectra. Therefore, the presence of extra nuclear RNA is probable in the nucleus as well as in the cytoplasm.

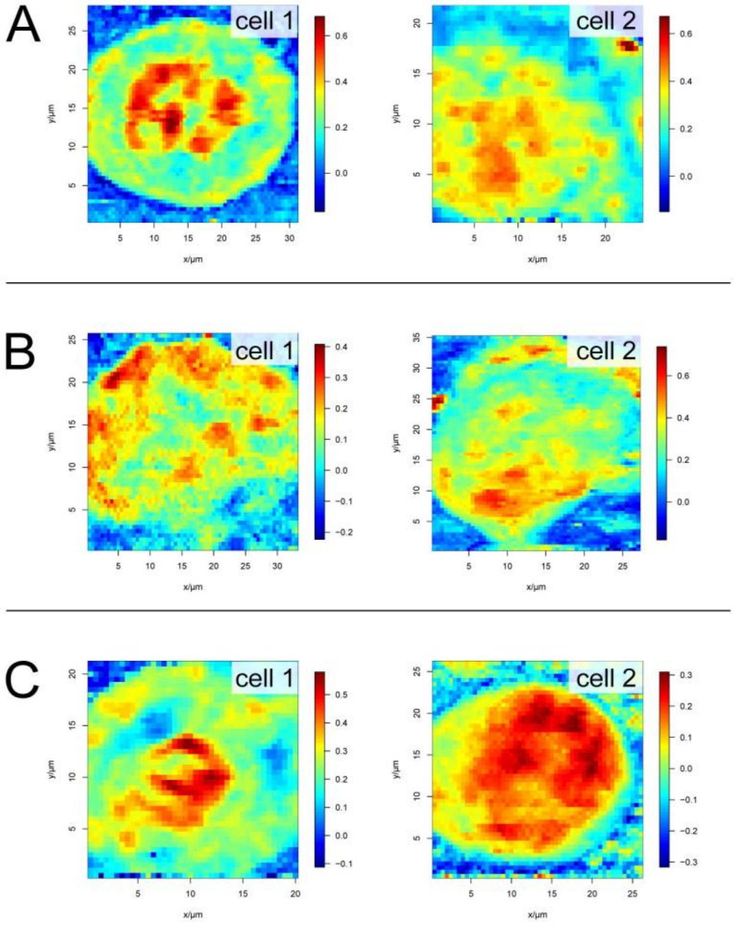

Figure S2: Chemical maps of the DNA/ RNA computation of MCF-7 cells by using ANN analysis are given. The cells were exposed to equal DCT concentration of 100 nmol/l for various exposure times: (A) 24 h, (B) 48 h and (C) 24 + 24 h. While almost all cells treated with 100 nmol/l show a fragmented nucleus, the second cell in C do not. This is probably caused by an individual behavior and the non-synchronized cell cycles of the cells
